# Pharmacodynamic and Pharmacokinetic Interactions of Propranolol with Garlic (*Allium sativum*) in Rats

**DOI:** 10.1093/ecam/neq076

**Published:** 2011-06-16

**Authors:** Syed Mohammed Basheeruddin Asdaq, Mohammed Naseeruddin Inamdar

**Affiliations:** ^1^Department of Pharmacology, Krupanidhi College of Pharmacy, Varthur Hobli, Chikkabellandur Village, Carmalaram Post, Bangalore 560 035, India; ^2^Department of Pharmacology, Al-Ameen College of Pharmacy, Bangalore, India

## Abstract

Garlic preparations and propranolol (PRO) are agents recognized as cardioprotective and potent antihypertensive agents when they are used individually. However, there is no report available to explain the role of combined therapy during simultaneous hypertension and myocardial damage in rats. We aimed to determine the pharmacokinetic and pharmacodynamic interaction of PRO with garlic homogenate (GH), in rats. The influence of garlic on pharmacokinetics of PRO was determined by HPLC method; while pharmacodynamic interaction was studied in animals with hypertension (10% fructose) and myocardial damage (isoproterenol, 175 mg kg^−1^, s.c. 2 days). PRO was given orally at 10 mg kg^−1^ for 1 week, whereas, GH was administered at three different doses of 125, 250 and 500 mg kg^−1^, p.o. in their respective groups during fourth to sixth week of high fructose (HF) period, once daily. Systolic blood pressure (SBP), heart rate, cholesterol, triglycerides, glucose, creatine phosphokinase-MB, lactate dehydrogenase, superoxide dismutase and catalase were measured and histopathological studies were carried out. The bioavailability and half life of PRO were significantly enhanced by 2- and 3-fold, respectively, in animals pretreated with garlic (250 mg kg^−1^). Administration of PRO and low to moderate doses of GH (125, 250 mg kg^−1^), either alone or together showed fall in fluid intake and body weight. The combined therapy of GH 250 mg kg^−1^ and PRO was found to be most effective in reducing SBP, cholesterol, triglycerides and glucose. These observations suggest that careful addition of garlic in moderate doses in PRO regimen might result in beneficial effect during treatment of hypertensive animals with myocardial damage.

## 1. Introduction

Currently reliance on natural products is gaining popularity to combat various physiological threats including oxidative stress, cardiovascular complexities, cancer insurgence, neurological disorders and immune dysfunction. The use of traditional remedies may be encountered more frequently due to an array of scientific evidence in their favor [1]. While such products had been used with apparent safety in traditional societies for many centuries, their combination with modern medicine, poses the possibility of potential interaction between the two groups of substances. Reports indicate that *∼*15–20% of individuals on prescription medications also use herbal supplements and <40% of patients disclose to their physicians the usage of herbal remedies, even if they experience severe side effects—because of the fear of censure or rebuke [2]. The problem is further compounded by the fact that many physicians are themselves not always familiar with the potential for herb-drug interactions [3]. Hence it is imperative to promote credible research on the safety and efficacy of combined herb-drug treatment for variety of ailments including cardiovascular diseases [4].

Propranolol (PRO) is a non-selective beta-blocker, that is, it blocks the action of epinephrine on both *β*_1_- and *β*_2_-adrenergic receptors. Despite complete absorption, PRO has a variable bioavailability due to extensive first-pass metabolism. Hepatic impairment and co-administration with food appears enhance its bioavailability [5].

Traditionally, garlic (*Allium sativum*, family: *Lilliaceae*) and its variety of preparations are widely used as therapeutically effective medicament for cardiovascular diseases. Consumption of garlic and cardiovascular disease progressions is inversely correlated [6]. The preparations of garlic have been widely recognized as agents for prevention and treatment of cardiovascular and other metabolic diseases such as atherosclerosis, arrhythmia, hyperlipidemia, thrombosis, hypertension and diabetes [7]. Furthermore, garlic is also reported to possess cardioprotective [8], antioxidant [9], antineoplastic and antimicrobial properties [10]. Furthermore, garlic has significant antiarrhythmic effect in both ventricular and supraventricular arrhythmias [11]. It is reported that chronic use of garlic in moderate doses augments the endogenous antioxidants activities and depletes the oxidative damaging effects by either increasing the synthesis of endogenous antioxidants or decreasing the generation of oxidants like oxygen free radicals [12]. Moreover, garlic also exerts anti-oxidant effect during isoprenaline-induced myocardial infarction in rat [13].

We recently reported improved survival and cardiac function by add-on captopril [14, 15] and hydrochlorothiazide [16, 17] during garlic therapy in rats with myocardial infarction. Our earlier experiment also demonstrated increased therapeutic efficacy of combined therapy of PRO with garlic in animals subjected to myocardial damage [18]. However, there is no scientific report to indicate the biochemical alterations, antioxidant profile, electrocardiographic parameters, hemodynamic findings and histological changes upon addition of PRO during chronic therapy of garlic in hypertensive rats with myocardial damage. Also, there is dearth of report on influence of garlic on pharmacokinetic parameters of PRO in rats. Hence, the present study was undertaken to evaluate the pharmacokinetic and pharmacodynamic interactions of PRO with garlic in rats.

## 2. Methods

### 2.1. Experimental Animals

Laboratory bred female Wistar albino rats (200–250 g) were housed at 25 ± 5°C in a well-ventilated animal house under 12:12 h light dark cycle. The rats had free access to standard rat chow (Amrut Laboratory Animal Feed, Maharashtra, India) containing (% w/w) protein 22.10, oil 4.13, fiber 3.15, ash 5.15, sand (silica) 1.12 and water *ad libitum*. The institutional animal ethics committee approved the experimental protocol and animals were maintained under standard conditions in an animal house approved by Committee for the Purpose of Control and Supervision on Experiments on Animals (CPCSEA).

### 2.2. Preparation of Garlic Homogenate

Garlic (*A. sativum*, family: *Lilliaceae)* bulbs were purchased from the local vegetable market. The cloves were peeled, sliced, ground into a paste and suspended in distilled water. Three different concentrations of the garlic homogenate (GH) were prepared, 0.05, 0.1 and 0.2 g ml^−1^, corresponding to 125, 250 and 500 mg kg^−1^ body weight of animal [12]. GH was administered within 30 min of preparation.

### 2.3. Fructose-Induced Hypertension

Seventy-two female Wistar rats were divided into nine groups of eight animals each. The group I was considered as control, given ordinary drinking water ad libitum throughout the whole treatment course and the remaining groups were given 10% fructose solution to drink ad libitum [19]. Three weeks later, the fructose-treated animals were assigned the following treatment regimens: group II: fructose-fed (HF), group III: fructose plus PRO (10 mg kg^−1^, p.o.) [20] in the sixth week, groups IV, V and VI were fed with fructose plus GH 125, 250 and 500 mg kg^−1^, respectively orally for 3 weeks, groups VII, VIII and IX were continued with fructose plus GH 125, 250 and 500 mg kg^−1^, respectively orally for 3 weeks as well as PRO (10 mg kg^−1^, p.o.) in the sixth week. Fluid/food intake, body weight, heart rate and systolic blood pressure (SBP) were measured every week. Concentrations of glucose, cholesterol and triglycerides [21, 22] were also measured in plasma samples at the end of the 6 weeks of high fructose.

### 2.4. Isoproterenol-Induced Myocardial Damage

At the end of treatment and recordings as mentioned above, animals of all groups except group I were administered isoproterenol (ISO) (175 mg kg^−1^ s.c.) for 2 consecutive days. Blood was withdrawn from retroorbital vein 48 h after the first dose of ISO under anesthesia and serum was separated by centrifugation for lactate dehydrogenase (LDH) and creatine phosphokinase-MB (CK-MB) measurement. The blood pressure and ECG changes were recorded under appropriate conditions. The heart was immediately isolated from each animal under ketamine (70 mg kg^−1^, i.p.) and xylazine (10 mg kg^−1^, i.p.) anesthesia. In each group consisting of eight animals, four excised hearts were homogenized to prepare heart tissue homogenate (HTH) using sucrose (0.25 M) [23]. The activity of LDH, CK-MB, superoxide dismutase (SOD) [24] and catalase [25] were measured in HTH. Microscopic slides of myocardium were prepared for histopathological studies from the hearts of remaining four animals. The myocardial damage was determined by giving scores depending on the intensity as follows [26]; no changes—score 00; mild—score 01 (focal myocytes damage or small multifocal degeneration with slight degree of inflammatory process); moderate—score 02 (extensive myofibrillar degeneration and/or diffuse inflammatory process); marked—score 03 (necrosis with diffuse inflammatory process).

### 2.5. Blood Pressure and Electrocardiograms Measurement

As discussed above, 48 h after first dose of ISO administration just prior to collection of blood samples, mean arterial blood pressure was measured in awaked animals by the non-invasive blood pressure module (NIBP pressure meter, LE 5001, V02/0402L, Panlab, Hardvard apparatus, Barcelona, Spain) and ECG was recorded in anesthetized animals (ketamine (70 mg kg^−1^, i.p.) and xylazine (10 mg kg^−1^, i.p.)) by subcutaneous lead II method (Physiograph, EKG coupler, SO-02, INCO, India), QRS duration, RR interval and QT segment were measured.

### 2.6. Statistical Analysis

Results of pharmacodynamic parameters are expressed as mean ± SEM. The statistical significance was determined using one-way analysis of variance (ANOVA) followed by Bonferroni's test. The results were considered statistically significant when *P* < .05.

### 2.7. Pharmacokinetic Interaction

Moderate dose of GH (250 mg kg^−1^, p.o.) was selected for pharmacokinetic interaction study as they were found to be more effective during pharmacodynamic evaluation. Animals were divided into two groups consisting of eight animals each: group I; PRO (PRO) 10 mg kg^−1^ p.o. (single dose) and group II; GH 250 mg kg^−1^ for 30 days (p.o) + PRO (single dose). Immediately after PRO administration, 0.5 ml of blood samples was withdrawn over 24 h (0, 0.5, 1, 2, 4, 8, 16 and 24 h) by puncturing retro orbital vein under ether anesthesia and subjected to analysis. The hypovolemia is prevented by intraperitoneal administration of 0.5 ml of normal saline immediately after each withdrawal of blood.

### 2.8. Extraction Procedure

The extraction buffer was 0.2 mol l^−1^ sodium carbonate, prepared by dissolving 21.2 g of sodium carbonate in 1 l of distilled water. The extraction solvent was butanol/hexane (20/80 by volume), both “HPLC” grade. Ammonium sulphate buffer (10 mmol l^−1^) was prepared by dissolving 1.32 g of ammonium sulphate in 1 l of doubly distilled water, then filtering it through a 0.45 *μ*m (pore size) Millipore filter and adjusting the pH to 6.8. After 100 *μ*l of serum is mixed with 200 *μ*l of the internal standard solution [*N*-(2-piperidylmethyl)-2,3-*bis*(2,2,2-trifluoroethoxy)-benzamide HCl, 2500 *μ*g l^−1^] and 200 *μ*l of 0.2 mol l^−1^ sodium carbonate, the sample is extracted with butanol/hexane (20/80 by volume). The organic layer is separated and evaporated, and the residue is redissolved in 200 *μ*l of methanol; 50 *μ*l of this is injected onto the column [27].

### 2.9. Chromatographic Conditions

The mobile phase for PRO (PRO) was composed of acetonitrile (solvent A) and phosphate buffer (solvent B), with 0.2% (w:v) of triethylamine, with the pH adjusted to 3 with orthophosphoric acid 85% (0.067 M, 40 : 60 v : v, pH 3). The flow rate was 0.8 ml min^−1^. The UV detection was accomplished at 294 nm at 0.05 AUFS and 0.5 s response time. Standard solutions of active principle were obtained by suitable dilution from stock solutions prepared at 0.25 mg ml^−1^ in phosphate buffer (pH 7.4, 0.067 M). The concentration range of the calibration curves was 1–8 *μ*g ml^−1^. The limits of quantitation were also determined by suitable dilution from the lowest concentration of the calibration curve range [28].

### 2.10. Pharmacokinetic Determination

For the generated data on PRO and garlic interaction to be analyzed, we assume that the kinetics of PRO elimination was linear [29]. The data was represented in a plasma level-time curve from where the area under time curve (AUC) was calculated using Trapezoid rule. The maximum concentration (*C*_max_) and maximum time (*T*_max_) were obtained directly from generated data. The elimination constant (*K*_e_ **)** and half life (*T*_1/2_) were determined from the semi-log plot of the data. The clearance (CL) and apparent volume of distribution (*V*_d_) of the drug in the animals were calculated from the equations, CL = *V*_d_ × *K*_e_, *V*_d_ is the administered dose of drug/initial plasma concentration of drug obtained at intercept of semi log plot of plasma drug sample. The mean plasma concentration-time curve for PRO (10 mg kg^−1^) alone and PRO plus once a day administration of oral GH (250 mg kg^−1^) for 30 days was determined. The results were analyzed statistically using Student's *t*-test.

## 3. Results

### 3.1. Fluid Intake, Food Intake and Body Weight

**Figure 1:**
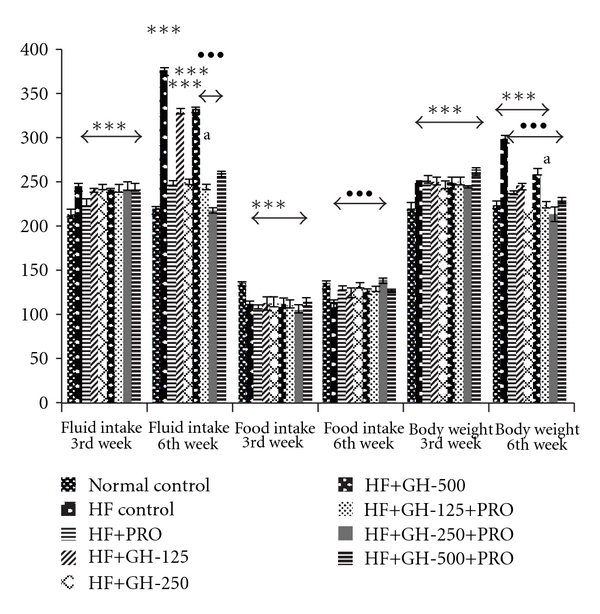
Fluid intake (ml kg^−1^ day^−1^), food intake (g kg^−1^ day^−1^) and body weight (g) in rats. All values are mean ± SEM, *n* = 8, ***P < .001 when compared to normal control; ^•••^ *P* < .001 when compared to HF control; ^a^ *P* < .05 when compared to PRO (comparison between PRO versus PRO + GH); HF, high fructose; PRO, propranolol, 10 mg kg^−1^, p.o. GH-125, 250 and 500, garlic homogenate 125, 250 and 500 mg kg^−1^, p.o. fluid intake (mg kg^−1^, p.o.) fluid intake (mg kg^−1^ day^−1^); food intake g kg^−1^day^−1^ body weight (g).

Fluid intake, food intake and body weight of the various groups of rats at the end of 3 weeks and 6 weeks are shown in Figure 1. High fructose for 3 weeks caused significant increase in fluid intake as well as body weight with decrease in food intake when compared to normal control. Treatment of animals with PRO and GH (250 mg kg^−1^), alone or together, found to bring back the normal conditions in fluid intake, body weight and food intake, to significant extent. The moderate dose of GH (GH 250 mg kg^−1^, p.o. for 3 weeks) was found to be most effective in ameliorating the abnormally increased fluid intake and body weight as well as decreased food intake compared to HF control. Although, GH at low and high doses (125 and 500 mg kg^−1^, resp.) demonstrated significant fall in fluid intake compared to HF control, but values were still significantly high when compared with normal control. Incorporation of PRO in the sixth week of HF intake shows synergistic effect in reversing the normalcy in fluid intake, food intake and body weight when compared to HF control. High and low doses of GH with or without PRO were less effective, whereas, moderate dose of GH was found to be most effective alone as well as along with PRO. 

### 3.2. SBP, Heart Rate, Cholesterol, Triglycerides and Glucose

**Figure 2:**
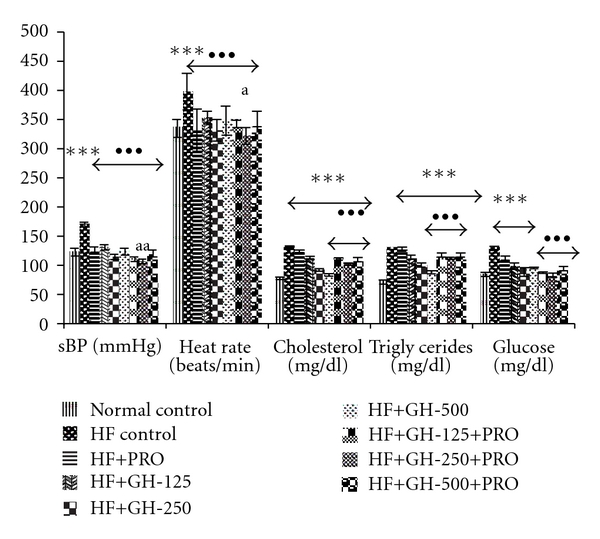
SBP (mmHg), heart rate (beats min^−1^), cholesterol (mg dl^−1^), triglycerides (mg dl^−1^) and glucose (mg dl^−1^) in rats. All values are mean ± SEM, *n* = 8, ***P < .001 when compared to normal control; ^•••^ *P* < .001 when compared to HF control; ^a^ *P* < .05, ^aa^ *P* < .01 when compared to PRO (comparison between PRO versus PRO + GH); HF, high fructose; PRO, propranolol, 10 mg kg^−1^ p.o. GH-125, 250 and 500, garlic homogenate 125, 250 and 500 mg kg^−1^, p.o.

The changes in SBP, heart rate, cholesterol, triglycerides and glucose in all the groups at the end of 6 weeks of high fructose in fluid are given in Figure 2. Six weeks of HF in water resulted in significant elevation in SBP, heart rate, cholesterol, triglycerides and glucose compared to normal control. Administration of PRO and various doses of GH demonstrated significant fall in all parameters to varying degrees when compared to HF control. The increased levels of blood pressure, heart rate, cholesterol, triglycerides and glucose were significantly lowered in animals treated with PRO (for 7 days during the sixth week of HF intake) compared to HF control. The maximum protective effect was seen in combination therapy of GH (250 mg kg^−1^, p.o. from fourth to sixth week of HF intake) with PRO (PRO, 10 mg kg^−1^, p.o. during the sixth week of HF intake) when compared to HF control. 

### 3.3. Electrocardiographic Parameters and Hemodynamic Findings

**Figure 3:**
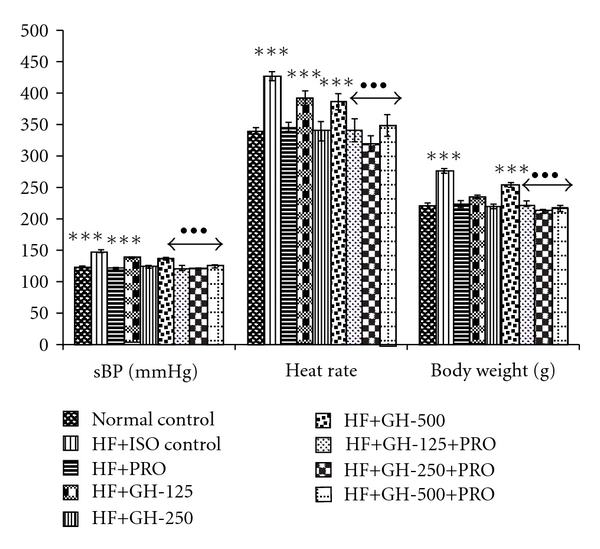
LDH activity and hemodynamic findings in rats. All values are mean ± SEM, *n* = 4/8, **P < .01  ***P < .001 when compared to normal control; ^•••^ *P* < .001 when compared to HF control; ^a^ *P* < .05 when compared to PRO (comparison between PRO versus PRO + GH); HF, high fructose; PRO, propranolol, 10 mg kg^−1^ p.o. GH, 125, 250 and 500, garlic homogenate 125, 250 and 500 mg kg^−1^, p.o.

**Figure 4:**
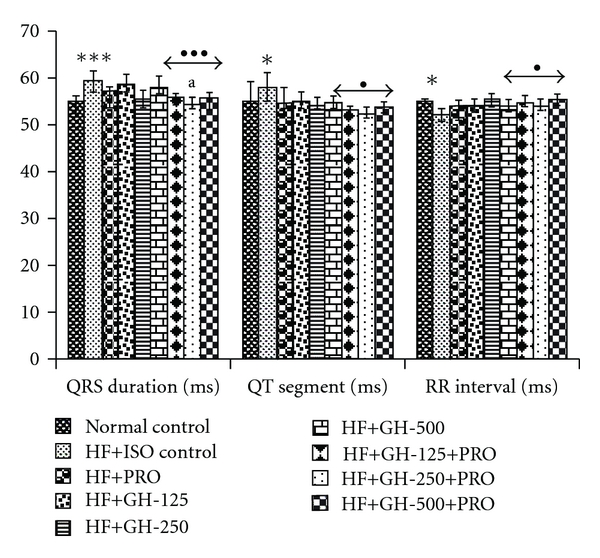
Electrocardiographic parameters. All values are mean ± SEM, *n* = 8; *P < .05, ***P < .001 when compared to normal control; ^•^ *P* < .05, ^•••^ *P* < .001 when compared to HF control; ^a^ *P* < .05 when compared to PRO (comparison between PRO versus PRO + GH); HF, high fructose; PRO, propranolol, 10 mg kg^−1^ p.o. GH, 125, 250 and 500, garlic homogenate 125, 250 and 500 mg kg^−1^, p.o.

High fructose intake for 6 weeks with subsequent administration of ISO showed significant increase in SBP and heart rate compared to normal control (Figure 3). Treatment of animals with different doses of GH and PRO caused fall in SBP compared to HF control. The combination of GH 250 mg kg^−1^ with PRO showed more reduction in SBP compared to their individual treatments. Moreover, the chronotropic effect was significantly reduced in all treatment groups compared to HF + ISO control. The combination of GH 250 mg kg^−1^ with PRO was more effective than individual treatment in reducing inclined heart rate as compared to HF + ISO control. The increased body weight due to high fructose intake for 6 weeks plus two doses of ISO was significantly reduced in PRO and GH treated groups as compared to HF + ISO control group. However, GH 500 mg kg^−1^ alone continued to show increased body weight when compared to normal control, whereas, upon addition of PRO, normal weight was attained. Significant changes in the ECG configuration were seen in the rats with HF + ISO treatment such as prolongation of QRS duration with longer QT segment and reduced RR intervals (Figure 4). The PRO treated animals showed significant prolongation of QRS complex compared to normal control. The combination of GH 250 mg kg^−1^ with PRO was found to significantly decrease the QRS complex compared to their individual treatment. Fall in QT segment and normalization of RR interval was seen with GH 250 mg kg^−1^ alone or in presence of PRO compared to HF + ISO control. 

### 3.4. Biochemical Parameters, Antioxidants and Histological Scores

**Figure 5:**
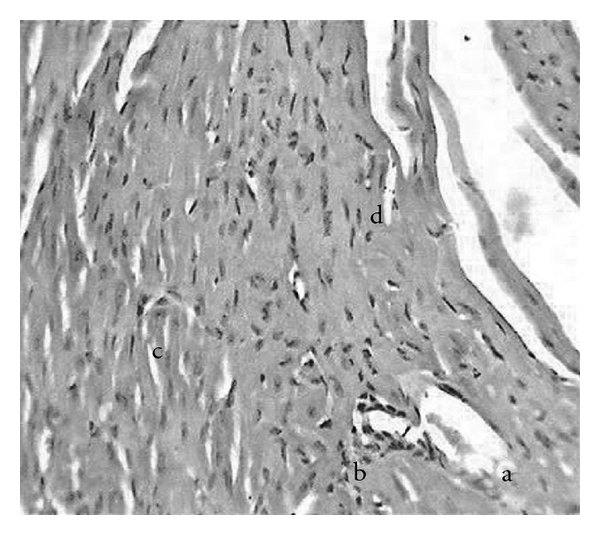
H&E (×400) stained microscopic section of high fructose (HF) plus isoproterenol (ISO) control. There is (a) loss of cellular architecture, (b) nuclear duplication and (c) increased infiltration of leucocytes with (d) prominent hyperchromasia.

**Figure 6:**
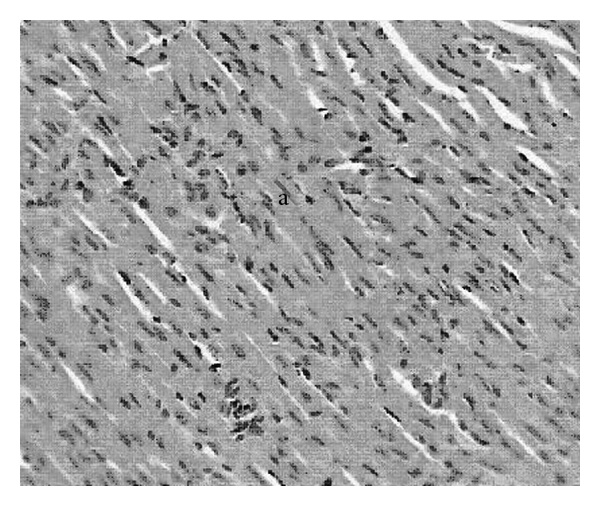
H&E (×400) stained microscopic section of heart tissue of animals pretreated with GH 250 mg kg^−1^ for 21 days orally. Normal cytoarchitecture of myocardium is seen (a).

**Figure 7:**
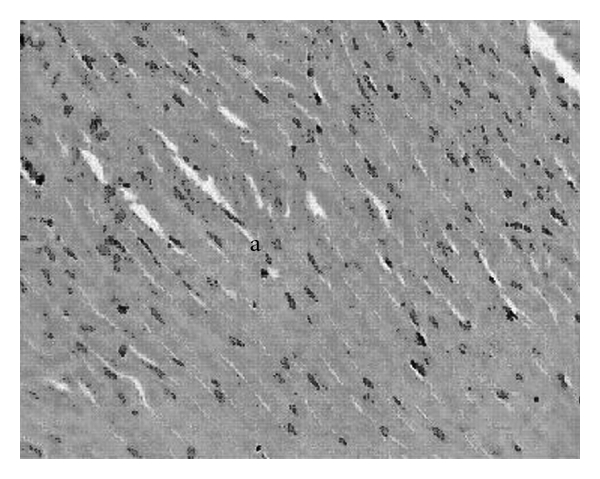
H&E (×400) stained microscopic section of heart tissue of animals pretreated with GH 250 mg kg^−1^ for 21 days orally and subsequently subjected to PRO 10 mg kg^−1^ orally for 7 days. Normal cytoarchitecture of myocardium with reduced interstitial space is evident from the figure (a).

High fructose fluid for 6 weeks and subsequent ISO administration for 2 days resulted in rise and fall in LDH and CK-MB (Table 1) activities in serum and HTH respectively, when compared to normal control. Oral administration of PRO or different doses of GH separately or together resulted in fall in LDH and CK-MB activities in serum and rise in HTH when compared to HF + ISO control. The best results were found with combination of PRO with GH 250 mg kg^−1^ per oral, as there was significant difference in CK-MB activity between GH 250 mg kg^−1^ alone and PRO + GH 250 mg kg^−1^. Table 1 also explains significant fall in SOD and Catalase activities with a rise in histological scores of animals subjected to high fructose intake plus ISO compared to normal control. The SOD and Catalase activities of all GH treated groups were found to be increased towards normal compared to HF + ISO control. However, incorporation of PRO during sixth week of high fructose failed to provide any incline in SOD and Catalase activities when compared to GH alone. The histological scores were found to be reverted to normal condition in all treated groups compared to HF + ISO control. There was loss of cellular architecture, nuclear duplication and increased infiltration of leucocytes with prominent hyperchromasia in animals subjected to high fructose fluid intake as well as ISO (Figure 5). Normal cytoarchitecture of myocardium was seen in animals treated with GH 250 mg kg^−1^ during the past 3 weeks of high fructose fluid intake (Figure 6). Microphotographs of animals provided protection of PRO as well GH 250 mg kg^−1^ shows normal cytoarchitecture of myocardium with reduced interstitial space (Figure 7). 

### 3.5. Pharmacokinetic Determination of PRO

**Figure 8:**
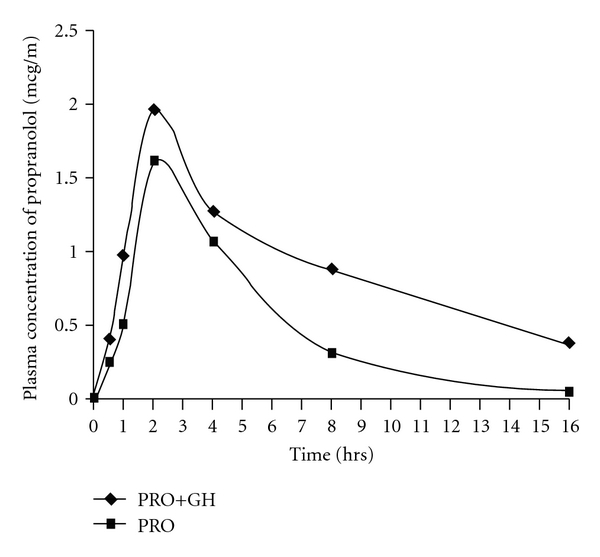
Plasma concentration of PRO in presence of garlic.

As depicted in Figure 8, *C*_max_ and AUC_total_ showed significant difference between the PRO alone and PRO + GH treated groups. The time to reach peak (*T*_max_) in the plasma concentration of PRO occurred at the same time in both groups, whereas *C*_max_ was substantially raised in presence of GH indicating increased extent of absorption. There was significant prolongation in elimination half-life *T*_1/2_ (h) in presence of GH from 2.32 to 6.6. The PRO clearance was also reduced significantly from 5.26 to 1.72 (ml kg^−1^ h) in presence of GH. It is also important to note that there was significant fall in the rate of absorption (*K*_a_) of PRO in presence of GH (from 1.92 to 0.42). Moreover, elimination rate of PRO when co-administered with GH was significantly lowered (Table 2). 

## 4. Discussion

The present study was undertaken to evaluate the pharmacokinetic and pharmacodynamic interaction of garlic with PRO using experimental models in rats. The results observed suggest that GH (250 mg kg^−1^, p.o.) when combined with PRO enhances the cardioprotective activity of latter during myocardial damage induced by ISO in rats. It also explains the ability of garlic to potentiate the antihypertensive actions of PRO in high fructose (HF) induced hypertensive rats. The bioavailability of PRO was remarkably increased when given to animals previously treated with GH (250 mg kg^−1^, p.o.).

Garlic (*A. sativum*) holds a unique position in history and was recognized for its therapeutic potential. Garlic used in the present study was purchased from the local vegetable market that is the most widely used form. Among thousands of published papers on the healing power of garlic, most of them employed fresh GH, as it is the most common consumable form for medicinal and non-medicinal purposes. The dose of garlic was selected based on dose-dependent study reported in earlier literature and later confirmed by our studies [14–18]. Garlic preparations contain a wide variety of organosulfuric compounds, *S*-allylcysteine (SAC), *N*-Acetylcysteine (NAC) [30] and *S*-allylmercaptocysteine (SAMC), which are mainly derived from alliin. When garlic tissue is disrupted, the enzyme alliinase comes into contact with alliin and catalyzes its breakdown into allicin [31–34]. Fresh GH is known to possess the highest concentration of active constituent, allicin with half life up to 2.4 days when compared to normal half life of allicin, 2–16 h [35, 36]. Allicin (allyl 2-propenethiosulfinate) was earlier thought to be the principle bioactive compound responsible for the cardioprotective effect. However, recent studies suggest that allicin is an unstable and transient compound with oxidant activity [37] that is virtually undetectable in blood circulation after garlic ingestion and decomposes to form the SAC and SAMC [37] by reacting with an enzyme allinase or alliin lyase, which is located only in the vascular bundle sheath cells [35]. GH was administered orally for 3 weeks to avail bioactivity of SAC and SAMC at highest level. The interaction and counteraction could be attributed to the interference of garlic in pharmacokinetic profile of conventional drug, PRO, PRO. Hence, the present investigation was undertaken to demonstrate the alterations in pharmacokinetics and pharmacodynamic of PRO, if any, in animals subjected to chronic prophylactic administration of GH.

Oral PRO administration has poor bioavailability due to extensive first-pass metabolism. The bioavailability of PRO may be increased by the concomitant ingestion of food and during long-term administration of the drug. The observations from our study demonstrate the role of garlic in pharmacokinetics of PRO. The AUC and *C*_max_ of PRO were remarkably elevated in presence of GH indicating significant reduction in the first pass metabolism of PRO in presence of GH. Besides, reduced clearance and enhanced *T*_1/2_ of PRO was seen in serum of animals pretreated with GH. Our findings in the present research were in accordance with their pharmacodynamic interactions that were reported by us recently [18]. Even though the rate of absorption of PRO was significantly reduced in presence of GH, the *C*_max_ of PRO both in presence and absence of GH was found to attain at the same time. Despite high *K*_a_ value, PRO when administered alone was found to undergo massive first pass metabolism that might be the reason for increased elimination, whereas, GH pretreatment causes slow rate of absorption of PRO without effecting the time to reach *C*_max_. The resulting elevation in AUC with fall in elimination rate could be due to enzyme inhibitory role of GH or by affecting the hepatic blood flow. Hence the use of garlic during PRO therapy should be monitored carefully with proper dose adjustments.

High concentration of fructose in diet and fluid has been reported and proved to induce hypertension with insulin resistance, impaired glucose tolerance and hyperinsulinemia [38]. The use of 10% fructose in drinking water, for 3 weeks or longer appeared to be most suitable for rapid production of fructose-induced hypertension [39]. The antihypertensive ability of PRO was intact before and after induction of myocardial damage and found to be potentiated when garlic was administered prophylactically. The crucial role of free radicals in pathogenesis of ISO induced myocardial damage has been discussed by various studies [40, 41]. The pathophysiological changes following ISO administration are comparable to those taking place in human myocardial alterations [26]. ISO induced myocardial damage is associated with decreased endogenous antioxidants such as superoxide dismutase (SOD) and catalase in serum that are structurally and functionally impaired by free radicals resulting in damage to myocardium [42]. Inclination in endogenous antioxidant activities in HTH is indication for structural integrity and protection to the myocardium that is achieved by prior administration of GH. It is interesting to note the alteration in SOD is with concomitant fluctuation in catalase after prior treatment of animals with GH. Elevated activity of catalase in HTH is more beneficial than increase in SOD activity alone because without a simultaneous increase in catalase activity, increased SOD activity may lead to intracellular accumulation of H_2_O_2_ with detrimental effects [43]. One of our important finding of the present study was maintenance of integrity of myocardium without simultaneous rise in endogenous antioxidants level in groups treated with PRO. Hence protection to myocardium during ISO was not only due to elevation in antioxidants but also by scavenging/preventing the formation of OFRs. PRO causes decrease influx of calcium across the cell membrane leading to dephosphorylation of myosin light chain kinase. It deactivates voltage sensitive calcium channels in the heart via G_s_ mediated mechanism independent of cAMP concentration. Therefore pacemaker activity and conduction velocity are decreased with resultant increase in refractory period. As it is known that oxidative phosphorylation is a central site of reactive oxygen species production in the heart [44]. Therefore by dephosphorylation, generation of OFRs can be drastically reduced. On the basis of the present observation, it is speculated that PRO mediate cardioprotection without significantly elevating SOD or catalase activities in HTH but by scavenging ability towards OFRs.

The membrane of myocardium was kept intact in animals pre-treated with GH (250 mg kg^−1^, p.o.) and PRO as evident from elevated LDH and CK-MB activities in HTH with depleted activities in serum. Damage to cardiac musculature was also demonstrated and confirmed by histopathological scores. An increase in score is indicative of myocardial damage [45]. Membrane stabilizing agent, PRO, was found to decrease the histopathological scores alone as well as when given along with cardioprotective, herb, garlic, in moderate doses of 250 mg kg^−1^ orally. The electrocardiographic parameters and hemodynamic findings were normalized in combination therapy especially RR interval and heart rate was reverted to normal conditions as well as QRS duration was reduced indicating protection from myocardial arrhythmias induced by ISO. These results suggest the synergistic behavior of PRO during GH mediated cardioprotection.

Higher doses of garlic might be containing more amount of allicin. Normally, upon administration, allicin is metabolically converted into safe active substances, SAC and SAMC, which are found to be antioxidant. However, at high concentration, allicin might not completely get converted into these safe substances and hence we found marked disturbance in biochemical and histological parameters at higher doses [46, 47]. This is corroborating with number of studies on garlic juice and GH demonstrating injurious effect of high dose of garlic on various tissues like intestinal lining and stomach [48].

**Figure 9:**
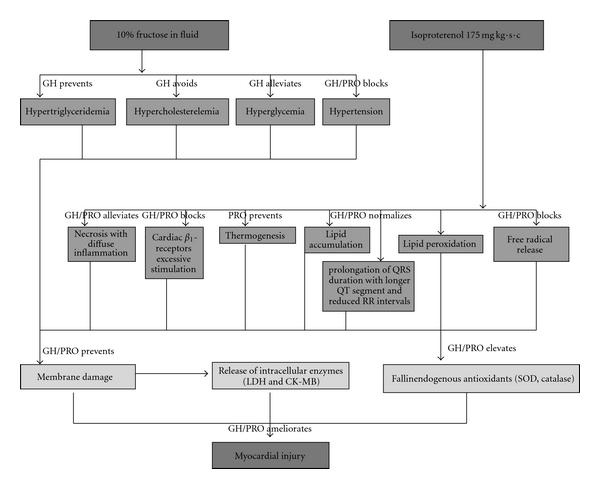
Mechanism of prevention of GH and PRO against fructose and isoproterenol induced hypertension and myocardial damage in rats. Both GH and PRO individually and together were able to inhibit the progression of myocardial necrosis by acting at various stages in the pathophysiology of hypertension and myocardial damage.

Hence from the above observations (Figure 9), the moderate dose of GH and/or PRO provided remarkable protection against high fructose in fluid (10% fructose)-induced hypertension by reducing triglycerides, cholesterol and hyperglycemia. The combined therapy also attenuated the ISO-mediated cardiac *β*_1_-receptors excessive stimulation [49], thermogenesis [50], myocardial hypoperfusion [51], glycogen depletion [52], electrolyte imbalance [53], lipid accumulation [54], lipid peroxidation [54], electrocardiographic disturbances [26] and free radical damage [55]. Therefore, it is possible that GH acts by enhancing the endogenous antioxidant defense system and PRO by curtailing the oxidative free radical generation, may maintain the myocardial membrane integrity and thus prevent cardiac dysfunction and hypertension. The results of the present study indicate that combining PRO with GH (250 mg kg^−1^, p.o.) could provide an opportunity to reduce the dose and frequency of PRO, which may help in minimizing repeated higher doses of PRO. 

## 5. Conclusion

In conclusion, this study revealed that garlic could cause increase in the bioavailability and half life along with decrease in the clearance and elimination rate constant of PRO when taken orally. This may pose a negative implication in clinical practice as toxicity of PRO may easily be reached especially during multiple dosing because of the possibility of drug accumulation. However, careful addition of garlic in moderate doses might result in beneficial effect during treatment of hypertension in animals with myocardial stress. Hence, further studies should be carried out to determine the influence of specific active constituent of GH when combined with PRO in animals subjected to hypertension and myocardial damage. We hope that this type of study will open new areas of research for interaction and counteraction between herb and conventional drugs when they are taken concurrently.

## Figures and Tables

**Table 1:** CK-MB, LDH, SOD, catalase activities and histological scores in rats.

Treatments	CK-MB activity		LDH activity		HTH		Histological scores
	Serum (U l^−1^)	Heart tissue homogenate (U g^−1^)	Serum (U l^−1^)	Heart tissue homogenate (U g^−1^)	SOD (U mg^−1^ protein)	Catalase (U mg^−1^ protein)	
Normal control	25.9 ± 2	44.1 ± 2	432 ± 5	626 ± 4	2.5 ± 0.0	3.2 ± 0.5	0.5 ± 0.2
HF + ISO control	38.4 ± 1.8***	21.2 ± 0.3***	565 ± 5***	386 ± 5***	1.5 ± 0.4***	1.7 ± 0.3***	2.6 ± 0.2***
HF + PRO	26.4 ± 3^∗•••^	38.3 ± 3^∗•••^	471 ± 4^••^	563 ± 2^•••^	1.9 ± 0.1^•••^	1.9 ± 0.2^∗••^	0.5 ± 0.2^∗••^
HF + GH-125	27.3 ± 1^∗••^	40.2 ± 2^•••^	415 ± 3^•••^	542 ± 3^•••^	2.2 ± 0.3^•••^	2.5 ± 0.4^∗∗••^	0.5 ± 0.2^•••^
HF + GH-250	25.2 ± 2^•••^	42.6 ± 1^∗•••^	421 ± 7^•••^	618 ± 9^•••^	2.4 ± 0.5^•••^	3.1 ± 0.3^∗•••^	0.5 ± 0.2^∗∗•••^
HF + GH-500	28.8 ± 1^∗••^	38.8 ± 1^∗•••^	459 ± 5^••^	531 ± 7^•••^	2.3 ± 0.1^•••^	2.8 ± 0.6^∗•••^	1.0 ± 0.3^∗••^
HF + GH-125 + PRO	25.2 ± 2^•••^	44.6 ± 3^•••^	446 ± 4^•••^	612 ± 6^•••^	2.3 ± 0.1^•••aa^	2.8 ± 0.4^•••aa^	0.5 ± 0.1^•••^
F + GH-250 + PRO	24.3 ± 3^•••^	46.4 ± 3^•••a^	441 ± 3^•••^	633 ± 7^•••^	2.4 ± 0.2^•••aa^	3.3 ± 0.2^•••aaa^	0.3 ± 0.2^•••^
HF + GH-500 + PRO	25.4 ± 5^•••^	44.5 ± 5^•••^	459 ± 7^•••^	613 ± 8^•••^	2.2 ± 0.1^•••aa^	2.9 ± 0.3^•••aa^	0.5 ± 0.1^•••^

All values are mean ± SEM, *n* = 4/8; HF—high fructose; PRO—propranolol, 10 mg kg^−1^, p.o. GH-125, 250 and 500—garlic homogenate 125, 250 and 500 mg kg^−1^, p.o.

*P < .05, **P < .01, ***P < .001 when compared to normal control; ^••^ *P* < .01, ^•••^ *P* < .001 when compared to HF control; ^a^ *P* < .05, ^aa^ *P* < .01, ^aaa^ *P* < .001 when compared to PRO (comparison between PRO versus PRO + GH).

**Table 2:** Pharmacokinetic parameters of PRO in presence and absence of GH.

Parameters	PRO	PRO + GH
*C*_ max_ (*μ*g ml^−1^)	1.63 ± 0.06	1.97 ± 0.07*
*T*_ max_ (h)	2.00 ± 0.00	2.00 ± 0.00
AUC_total_ (*μ*g h^−1^ ml^−1^)	7.98 ± 0.79	15.77 ± 1.34**
*K*_ e_ (h^−1^)	0.3231 ± 0.05	0.167 ± 0.01***
CL (ml kg^−1^ h^−1^)	5.26 ± 0.68	1.72 ± 0.20***
*T*_1/2_ (h)	2.32 ± 0.50	6.67 ± 0.79***
*V*_ d_ (ml kg^−1^)	16.71 ± 1.10	16.15 ± 0.23
*K*_ a_ (h^−1^)	1.32 ± 0.22	0.67 ± 0.09*

Values are mean ± SEM, *n* = 8; PRO means propranolol 10 mg kg^−1^; GH means Garlic homogenate 250 mg kg^−1^. In PRO group-single dose of PRO p.o. and in interactive group-30 days of GH treatment p.o. + single dose of PRO p.o.

*P < .05, **P < .01, ***P < .001 when compared to PRO alone.
